# Diagnosis and Management of Carpal Tunnel Syndrome in Children with Mucopolysaccharidosis: A 10 Year Experience

**DOI:** 10.3390/diagnostics10010005

**Published:** 2019-12-20

**Authors:** Ivana Dabaj, Cyril Gitiaux, Daniela Avila-Smirnow, Jacques Ropers, Isabelle Desguerre, Arielle Salon, Stéphanie Pannier, Abdellah Tebani, Vassili Valayannopoulos, Susana Quijano-Roy

**Affiliations:** 1 CHU de Rouen, Service de néonatologie, réanimation pédiatrique, neuropédiatrie et éducation fonctionnelle de l’enfant, 76000 Rouen, France; 2 APHP, Hôpital Raymond Poincaré, Hôpitaux Universitaires Paris Ile-de-France Ouest, Pôle pédiatrique, Service de Pédiatrie, 92380 Garches, France; 3 Centre de Reference Nord-Est-Ile de France pour le Réseau national de maladies neuromusculaires (FILNEMUS), 75015 Paris, Franceisabelle.desguerre@aphp.fr (I.D.); 4 Service de neurophysiologie clinique pédiatrique, Hôpital universitaire Necker Enfants Malades, APHP, Université de Paris, 75015 Paris, France; 5 Sección de Neurología, División de Pediatría, Facultad de Medicina, Pontificia Universidad Católica de Chile, Unidad de Neurología, Servicio de Pediatría, Complejo Asistencial Dr. Sótero del Río, Santiago 8330077, Chile; 6 Unité de recherche clinique, Hôpital Pitié Salpetrière, AP-HP, 75013 Paris, France; 7 Université Paris Descartes, Sorbonne Paris Cité. Service d’orthopédie infantile, Hôpital Necker enfants malades, 75015 Paris, France; 8 CHU de Rouen, laboratoire de biochimie métabolique, 76000 Rouen, France; 9 Sanofi Genzyme, 50 Binney Street, Cambridge, MA 02142, USA; 10 INSERM U1179, Université Versailles Saint-Quentin (UVSQ), UFR des sciences de la santé Simone Veil, 78180 Montigny, France

**Keywords:** carpal tunnel syndrome (CTS), electrodiagnostic studies (EDX, EMG), mucopolysaccharidosis (MPS), MNCV-w, DML, SNCV

## Abstract

Introduction: Mucopolysaccharidoses (MPS) are rare and clinically heterogeneous lysosomal storage disorders. Carpal tunnel syndrome (CTS) is a frequent complication in MPS types I, II, VI, and VII. CTS symptoms are difficult to recognize in these children, and often there is a lack of appropriate investigations. Patients and methods: In this retrospective study, all MPS patients were referred to the electrodiagnostic (EDX) laboratory of a single academic center during a 10-year period. Forty-eight children underwent serial EDX studies for CTS diagnosis and follow-up after surgery. Forty-two patients were diagnosed with CTS. Sensory nerve conduction velocity (SNCV), distal motor latency (DML), and motor nerve conduction velocity through the wrist (MNCV-W) of the median nerve were reviewed and analyzed. Results: One-hundred-three EDX examinations were performed on 48 patients. The median age at disease diagnosis was 2.1 years versus 4.9 years for CTS diagnosis. Analysis of the series revealed that electrophysiological abnormalities of CTS could have started much earlier (before the age of 2 years or at diagnosis of MPS). Diagnosis was based on SNCV and DML results, and MNCV-W was taken into consideration. Bilateral CTS was frequent (88%) in the types of MPS studied in our population and was observed from the first year of life, and may not have be associated with obvious clinical symptoms. EDX studies also helped in the follow-up and detection of CTS relapses, thus leading to an early intervention allowing a better recovery. Conclusion: EDX studies should be performed promptly and regularly in these patients. Prospective studies are required in order to understand the effect of disease-specific therapies in preventing the development of CTS in these patients. Synopsis: EDX studies should be performed in MPS patients soon after diagnosis and during routine follow-up, before and after surgical decompression.

## 1. Introduction

Mucopolysaccharidoses (MPS) are rare inherited lysosomal storage diseases resulting from a deficiency of one or more lysosomal enzymes involved in glycosaminoglycan (GAG) metabolism and subsequent abnormal accumulation of GAGs in cells, tissue, and organs [1,2]. MPS are multi-organ disorders and may affect the central and peripheral nervous system, heart and lungs, ear–nose–throat and upper airways, eyes, bones and joints. Carpal tunnel syndrome (CTS) and flexor tendon triggering are common orthopedic manifestations in this spectrum of disorders [2]. CTS is an entrapment neuropathy of the median nerve at the level of the carpal tunnel (CT), where the nerve resides with the flexor tendons [3]. While it is the most common entrapment neuropathy in adults (4–5% of the population especially between ages 40 and 60), it is extremely rare in children [3,4,5,6]. The most common etiology in the pediatric population is due to lysosomal storage diseases and more specifically, MPS [7]. Occasionally, a diagnosis of CTS in a child might indicate an MPS diagnosis [6,8]. In this study, we review the medical records in all MPS patients who were referred to our electrodiagnostic (EDX) laboratory during a 10-year period (2004–2014). We aim to describe the optimal age to refer a patient with MPS for a first EDX study, frequency of EDX studies, indications for surgery, and follow-up. At the neurophysiological level, our objectives are to detect the most useful and sensitive parameters to diagnose CTS. Our secondary aim is to study the impact of specific therapies for MPS such as enzyme replacement therapy (ERT) and hematopoietic stem cell transplant (HSCT), on the occurrence of CTS.

## 2. Patients and Methods

### 2.1. Patients

We included all patients with MPS that visited the neurophysiology department at Necker enfants malades university hospital. These patients were evaluated by EDX testing in our laboratory between 2004 and 2014. We reviewed the medical records and extracted the following information: date of birth, date of last follow-up, gender, type of MPS, date of diagnosis of MPS, subtype of MPS, molecular diagnosis of MPS; signs and symptoms related to CTS, cognitive delay, date of EDX test, date of diagnosis of CTS, date of surgery, type of surgery, unilateral or bilateral intervention; number of surgical decompression of the CT; HSCT: date of transplant, phenotype; ERT: date of initiation, dose, date of stopping the ERT.

### 2.2. Methods

Patients underwent EDX studies using a portable Keypoint machine. Before each study, their temperature was checked, and cool limbs (<32 °C) were warmed. Sensory nerve action potentials (SNAP) were recorded orthodromically using a ring electrode at the second digit for stimulus with the cathode proximal and the recording electrode over the wrist at the median nerve level. Distal latency, peak-to-peak amplitude of the SNAP, and sensory nerve conduction velocity (SNCV) were measured. Compound muscle action potentials (CMAPs) of the median nerve were also recorded and involved stimulation of the median nerve at the wrist and the palm, while the active recording electrodes were over *abductor pollicis brevis*. Distal motor latency (DML), peak to baseline amplitude of the CMAPs, and motor nerve conduction velocity through the wrist (MNCV-W) were recorded using standard procedures [8]. Raw data of SNCV, DML, and MNCV-w were compared to age-matched values of our normative laboratory data. Age-related normative data for median nerves were established using data from our laboratory (patients presented with no clinical signs in upper limbs and for those all the diagnostic workup was normal).

CTS diagnosis using EDX studies was defined by the following criteria: (1) decreased SNCV compared to our laboratory normative data or absent SNAP, (2) prolonged DML of the median nerve across the wrist when compared to our laboratory normative data or CMAP is absent. 

We used Loess polynomial regression to model the evolution of EDX parameters over time. Calculations were done using R software [9].

## 3. Results

### 3.1. Clinical Data

**Figure 1:**
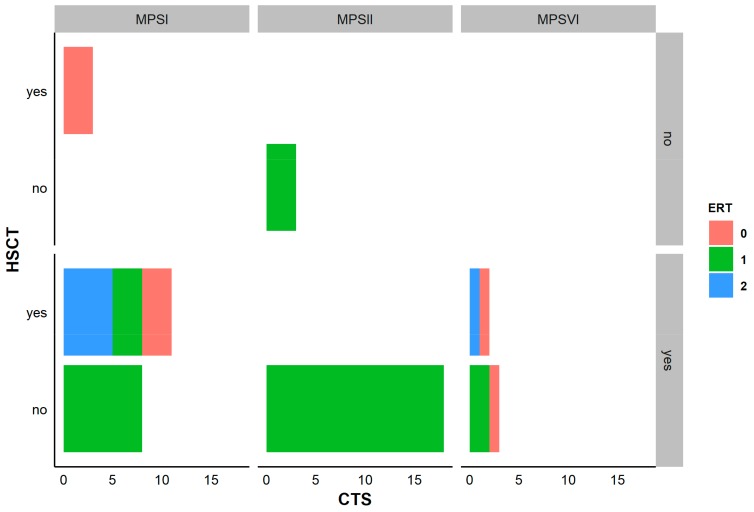
Specific therapies for MPS and treatment. Visualization of the explored specific therapies for MPS, including enzyme replacement therapy (ERT) and hematopoietic stem cell transplant (HSCT), along with the occurrence of CTS. ERT 0 = no treatment, 1 = chronic treatment, 2 = temporary treatment. CTS numbers correspond to the number of patients. In MPS I patients, we differentiate 3 groups: one which received HSCT without ERT and had no CTS, one treated exclusively with ERT where all had CTS, and one which received ERT temporarily or chronically or received no ERT where all had CTS. In MPS II patients, all of them took ERT and the majority have CTS. In MPS VI patients, all have CTS, whatever was the type of treatment for the disease.

In our study, there was a clear male predominance (38 boys, ten girls). The mean age of the patients in our cohort at the last follow-up was 9.84 years (median 8.6 years, range 1.88 to 20.16 years). In our population, we observed almost the same number of patients with MPSI and MPSII (21 MPSI and 22 MPSII) and five patients with the very rare type MPS VI (Figure 1, Supplementary Data).

When reviewing the symptoms and signs related to CTS, 20 patients did not report any symptoms, 14 were symptomatic, and for 14 patients, no information about symptoms were noted in the medical chart. All patients had hand deformities. Some of them had a loss of manual dexterity or intermittent, excessive crying at night that started a few months before EDX diagnosis of CTS and sometimes paresthesia all over the hand. A cognitive delay was noted in 75% of patients in our series in addition to behavioral disturbances; therefore the clinical signs of thumb weakness and impaired sensation were impossible to evaluate. Assessing thenar wasting and pulp atrophy was, in some cases, hard to be noted due to the combination of mental retardation and hand deformities.

Concerning the treatment, fifteen patients had received HSCT and 35 ERT; some patients were on concomitant therapy for MPS (Figure 1, Supplementary Data). In MPS I patients, those treated exclusively with ERT all had CTS. Among MPS I patients who underwent HSCT, 11 developed CTS. All MPS II patients were receiving ERT, 19 out of 22 have CTS. All patients with MPS VI in our series developed CTS, whatever was the treatment given (Figure 1).

### 3.2. Neurophysiology Results

The majority of the patients, 87% (42 out of 48) were diagnosed to have CTS. The time delay between MPS and CTS diagnosis varied between 1 and 83 months and one patient had CTS diagnosed one month prior to confirmation of MPS diagnosis (median = 28 months). Thirty-five out of forty-two (83%) of the CTS group had a surgical intervention on both hands. The median age at diagnosis of CTS was 4.6 years (20 months–14.6 years); the mean was 5.5 years and the SD 2.8 years. The median age at last follow-up was 8.8 years for MPS patients with CTS while patients without CTS at last follow-up were younger (median = 5.8 years). In 6 patients (3 MPS I: patients 2, 11, 12; and 3 MPS II: patients 23, 29, 42), no signs of CTS were detected. Interestingly, the three MPS I patients who did not develop CTS were treated for HSCT before the age of 2 years and they did not show CTS by the age of 2.5, 16.1, and 16.5 years, respectively. Two patients with MPS I were treated early by HSCT and had normal EDX studies after 8 and 11 years of surgical decompression, thus not showing any relapse. MPS II patients had no CTS at 4, 5, and 7 years (Supplementary Data).

### 3.3. Preoperative Results

Forty-eight EDX studies were performed preoperatively in 32 patients (20 patients had 1 EDX study performed, 8 had 2 EDX studies, and 4 had 3 EDX studies preoperatively). Twenty-six were diagnosed to have CTS based on EDX testing. Seven EDX tests were normal in 5 patients before the diagnosis of CTS. CTS was bilateral in 22/25 (88%) of the patients who had undergone preoperative studies, but this percentage may be underestimated because not all patients had bilateral examination due to technical difficulties or patient refusal.

**Figure 2:**
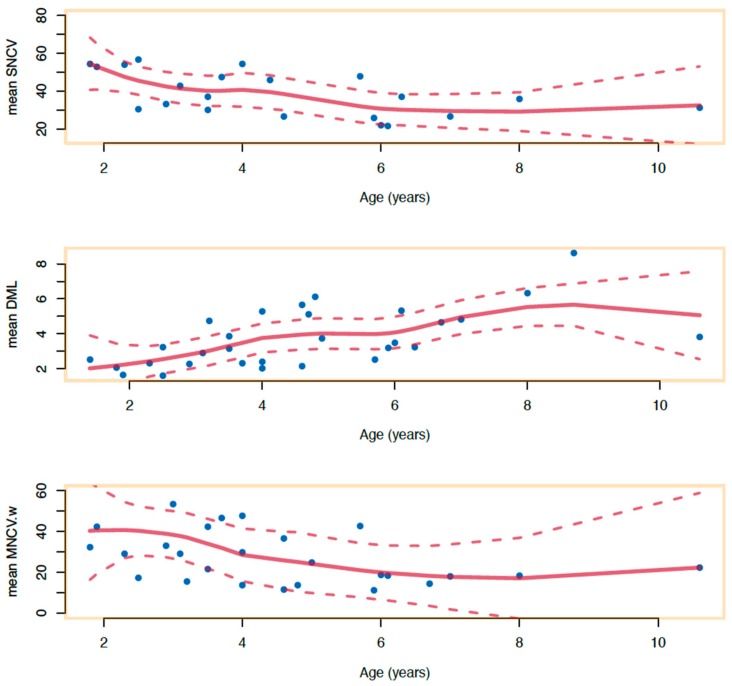
Evolution of preoperative values of SNCV, MNCV-w, DML with age: the progression of the electrophysiological abnormalities with age. Loess non-parametric regression shows that SNCV and MNCV-w decrease while DML increases with age.

**Figure 3:**
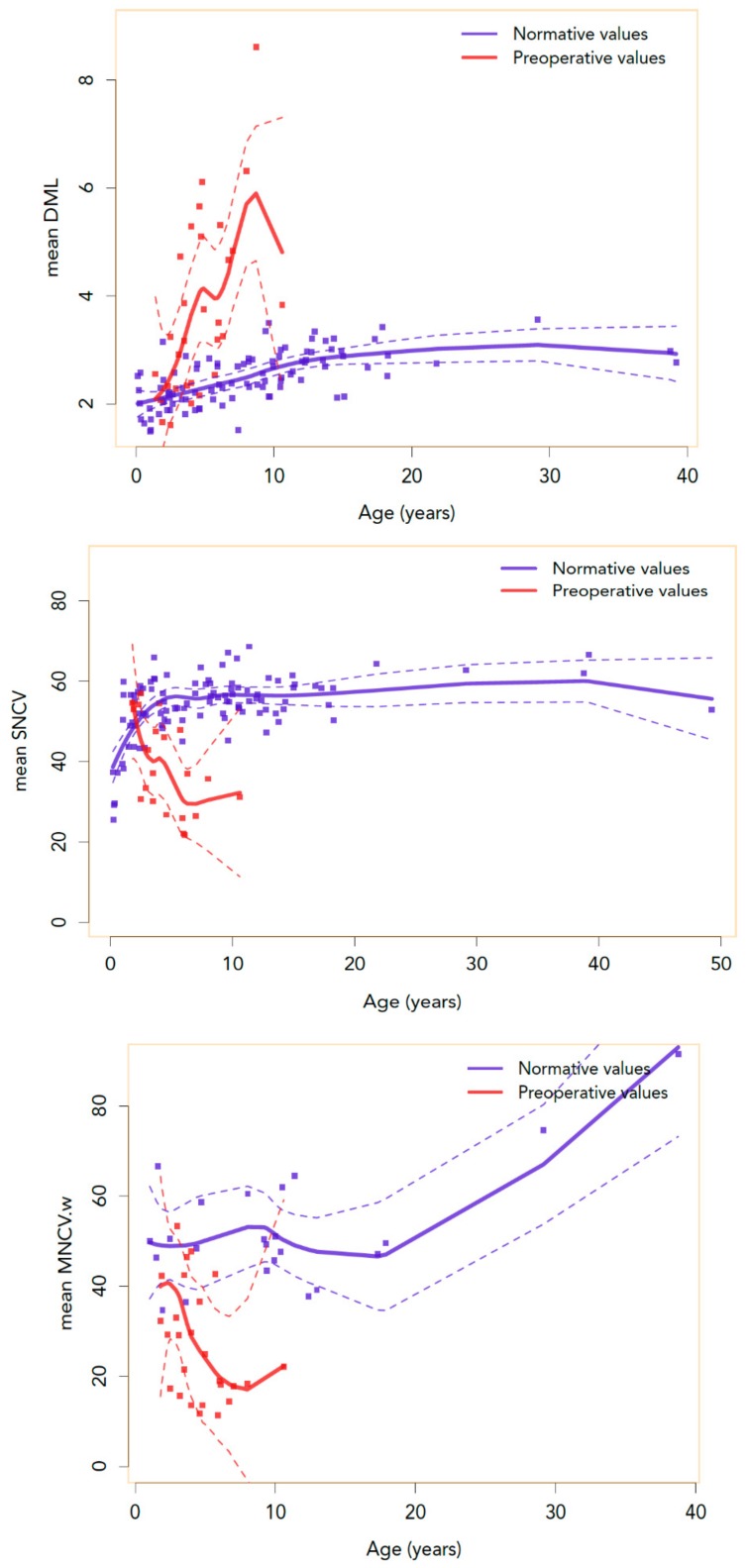
Normative and preoperative values of SNCV, MNCV-w, DML with age. The curves represent the Loess non-parametric regression normative and preoperative values of SNCV, MNCV-w, and DML with age. There is an intersection between normative and preoperative values of SNCV at 26 and 17 months for DML, suggesting that electrophysiological abnormalities appear around these ages.

**Figure 4:**
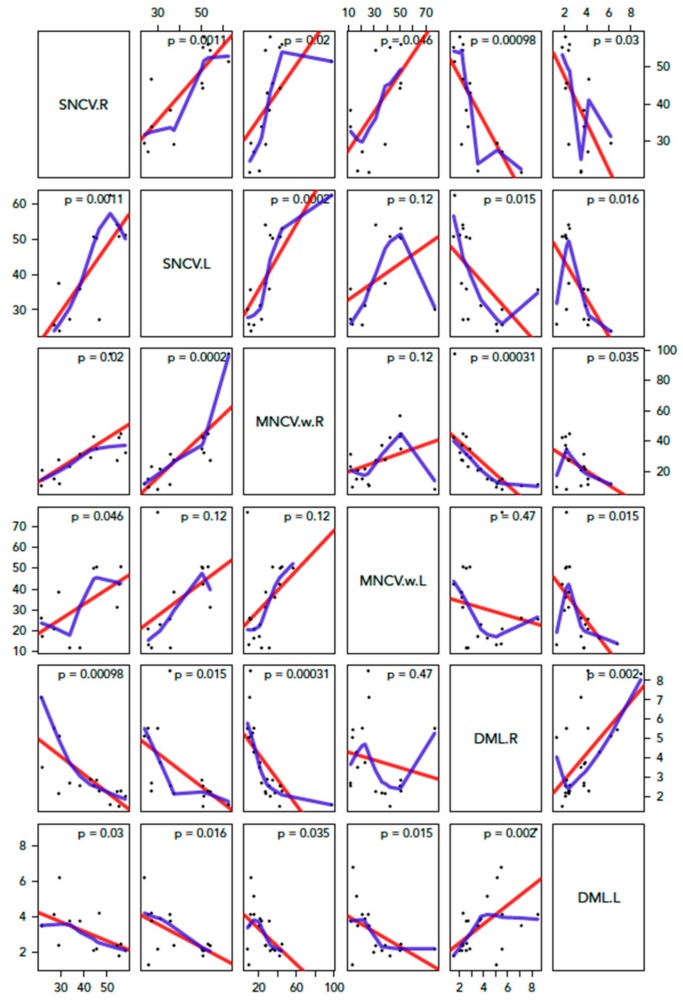
Correlation between SCNV, MNCV-w, DML. There is a negative correlation between DML, SNCV, and MNCV-w. We also explored whether a difference exists between right and left hand but the results were comparable.

The average age on the last EDX study before surgery was 4.7 years. When studying the cases with initial normal EDX test and development of CTS at follow-up, a negative correlation is found between age and median SNCV through the wrist. Additionally, prolongation of mean DML through the wrist is found to increase with time (Figure 2). When comparing these curves with the curves of our normative data, abnormalities seem to appear around the age of 26 months using SNCV and by 17 months using DML (Figure 3). Furthermore, we had systematically recorded MNCV-W and the curves obtained are highly correlated with SNCV and DML findings (*p*-value < 0.001; Figure 4).

### 3.4. Postope Rative Results

Fifty-five EDX studies were performed after surgical decompression in 30 patients (15 patients had 1 EDX test performed, nine had 2 EDX studies, three had 3 EDX studies, two had 4 EDX studies and one had 5 EDX studies done postoperatively). Most patients with CTS had surgery (35/42; 83%). Surgery was performed under general anesthesia. The median age at first surgical decompression was 4.8 years (range 2 to 14 years, mean 5.7 years). Fifteen patients had their first surgical decompression before their first EDX test (usually concomitant with other surgeries or because patients were symptomatic). In the remaining 20 patients, there was a delay between the diagnosis of CTS and the surgery ranging between 1 month and 4.4 years (median = 3 months).

A progressive improvement of median nerve SNCV and DML values was noted post-operatively, but studies performed before 6 months did not show full recovery. Six months after surgery, EDX testing in five patients was improved or was normal. At one year, six patients showed normalization of DML only, and sometimes of all parameters. At two years, all parameters were improved or normalized in 23 patients. Twelve cases with only late postoperative EDX testing showed abnormal findings and it was not possible to determine if they were due to residual abnormalities following an incomplete recovery or a relapse (no earlier post-operative baseline studies were available).

Five of the operated patients (two MPS type VI, two MPS type I-S, one MPS type II) showed a relapse of CTS (14% of those operated). Surgical decompressions were performed twice in three of these patients (second surgery was done after 1 to 11 years; Supplementary Data).

## 4. Discussion

This study provides a description of EDX features in a large population of 48 children with MPS. SNCV was the most sensitive parameter in preoperative studies for the detection of borderline diagnoses, for early diagnosis of CTS and follow-up before and after surgery. DML also improved after surgical decompression.

CTS is very common in patients with MPS, but the age of diagnosis depends on the age when EDX testing was performed or when the CTS release surgery was done. In the series of Kwon et al., the authors reported CTS starting at age 26 months in 95% of patients in their group (MPS II patients exclusively aged between 4 and 408 months; [10]). In another study of 29 MPS I patients, the authors detected CTS in 66% of the cases [11], the same percentage detected in a series of patients with MPS I described by Thomas JA. et al. in 2010 [12]. In our series, it is present in 88% of patients and we assume that it starts around the age of 17 months. When we compare the curves of mean SNCVs in normal subjects with those of MPS patients, we note that 26 months is the age where MPS patients deviate from the normal controls, what may be interpreted as a mean “cut-off” age to expect the onset of the sensory neuropathy. Concerning DML, if we compare the DML of normal subjects versus MPS patients, we found a cut-off age of 17 months for the motor neuropathy. This means that we might have been able to detect CTS earlier in our series if the investigations had been done between 17 and 26 months of age or even earlier. Our limitation in this study is that only five patients had their EDX studies performed before 26 months, making it difficult to confirm our hypothesis. However, we recommend initiating electrophysiological testing at MPS diagnosis or before two years of age.

Kwon et al. defined CTS as prolonged median nerve sensory or motor distal latencies or decrease in conduction velocity across the wrist in addition to the absence of SNAP or CMAP [10]. We used median nerve SNCV and DML as diagnostic parameters of CTS in children with MPS (decrease SNCV or absence of SNAP, and/or prolongation or absence of DML). In our series, SNCV was the most sensitive parameter in preoperative studies for the detection of borderline cases, early diagnosis of CTS and follow-up before and after surgery. According to our experience, all MPS patients should undergo EDX testing despite presence or absence of clinical signs or symptoms because nerve responses could be absent at an early age and, therefore, there is a need to do early screening studies [13,14]. Muenzer et al. recommend yearly follow-up studies [15]. We suggest starting the first follow-up study at six months after the initial examination, then performing these investigations on a yearly basis even if the previous EDX result was normal. Bilateral examinations are advised. Our results suggest that the earlier the CTS is treated surgically, the better are the results. Therefore, more frequent visits should be scheduled if the electrophysiologic values are borderline (every six months), in order to detect the entrapment neuropathy as early as possible and perform a prompt surgical intervention. Postoperative follow up should take place at six months, one year, and then once per year. EDX testing should be performed not only to monitor the efficacy of the surgical treatment but also to be able to distinguish between a relapse and an incomplete recovery in the presence of an abnormal EDX study in the postoperative period. The first postoperative study should not be done before six months because recovery is progressive and often incomplete before this time. Conversely, a baseline study two years after surgery may be too late, not being possible to distinguish if the median nerve is in the process of recovering or if it is relapsing or not adequately treated.

As suggested by other authors, we recommend early surgical decompression of the median nerve [5,6,8,16,17]. When surgery was delayed, the recovery of electrophysiological signs was slow. The orthopedic surgeons performed an open approach in all our cases (open division of the flexor retinaculum) as in the series of Haddad et al. An endoscopic approach is not advisable in children and not used because the field of view is narrowed and does not allow inspection of the CT [5].

Therapies for MPS consist of ERT and HSCT [18]. ERT does not prevent the development of CTS [6]. Concerning HSCT, studies are controversial about the effect on CTS. Wraith and Alani mention that HSCT fails to prevent or reverse CTS in MPS patients because of the avascular nature of the flexor retinaculum making the penetrance of the donor enzyme difficult in the dense connective tissue [19]. Interestingly, Grigull et al. reported an improvement of CTS in one of the four cases that underwent HSCT without any surgical intervention [13]. When it comes to joint rigidity, Imaizumi et al. reported improvement of joint contractures after HSCT in MPS II and MPS VI [20]. Our results suggest that this therapy might be delayed or possibly prevent the occurrence of CTS in MPS I because two patients did not suffer from CTS, for as long as the age of 16 years (patients 11, 12).

## 5. Conclusions

During the past two decades, the outcome for patients with MPS disorders has improved considerably due to supportive care and the availability of specific treatment. Due to these advances, children with even the most severe forms of MPS are now living into adolescence or beyond, which prompt health professionals to provide them with the best care for the best functional outcome. CTS can be painful and disabling; therefore, early diagnosis and treatment should be provided. EDX studies should be performed promptly and regularly in these patients. Prospective studies are required in order to understand the effect of disease-specific therapies in preventing the development of CTS in these patients.

## Supplementary Materials

Supplementary materials can be found at https://www.mdpi.com/2075-4418/10/1/5/s1.
